# Anti-allergic and anti-inflammatory effects of butanol extract from *Arctium Lappa L*

**DOI:** 10.1186/1476-7961-9-4

**Published:** 2011-02-08

**Authors:** Eun-Hwa Sohn, Seon-A Jang, Haemi Joo, Sulkyoung Park, Se-Chan Kang, Chul-Hoon Lee, Sun-Young Kim

**Affiliations:** 1 Department of Herbal Medicine Resource, Kangwon National University, Samcheok, 245-711, Korea; 2 College of Pharmacy, Sungkyunkwan University, Suwon, 440-746, Korea; 3 Department of Natural Medicine Resources, Semyung University, Jecheon, 309-711, Korea; 4 College of Pharmacy, Hanyang University, Ansan, 426-791, Korea; 5 Department of Pediatrics, College of Medicine, Hanyang University, Seoul, 133-792, Korea

## Abstract

**Background:**

Atopic dermatitis is a chronic, allergic inflammatory skin disease that is accompanied by markedly increased levels of inflammatory cells, including eosinophils, mast cells, and T cells. *Arctium lappa L*. is a traditional medicine in Asia. This study examined whether a butanol extract of *A. lappa *(ALBE) had previously unreported anti-allergic or anti-inflammatory effects.

**Methods:**

This study examined the effect of ALBE on the release of β-hexosaminidase in antigen-stimulated-RBL-2H3 cells. We also evaluated the ConA-induced expression of IL-4, IL-5, mitogen-activated protein kinases (MAPKs), and nuclear factor (NF)-κB using RT-PCR, Western blotting, and ELISA in mouse splenocytes after ALBE treatment.

**Results:**

We observed significant inhibition of β-hexosaminidase release in RBL-2H3 cells and suppressed mRNA expression and protein secretion of IL-4 and IL-5 induced by ConA-treated primary murine splenocytes after ALBE treatment. Additionally, ALBE (100 μg/mL) suppressed not only the transcriptional activation of NF-κB, but also the phosphorylation of MAPKs in ConA-treated primary splenocytes.

**Conclusions:**

These results suggest that ALBE inhibits the expression of IL-4 and IL-5 by downregulating MAPKs and NF-κB activation in ConA-treated splenocytes and supports the hypothesis that ALBE may have beneficial effects in the treatment of allergic diseases, including atopic dermatitis.

## Background

Atopic dermatitis is a chronic, allergic inflammatory skin disorder characterized by pruritic chronic eczema, elevated serum IgE levels, and massive cellular infiltrates, including eosinophils, mast cells, and lymphocytes [1,2]. Because mast cells play essential roles in provoking the pathogenesis of allergic reactions via the degranulation process, measuring the degree of degranulation reflects the level of mast cell activation. β-Hexosaminidase released by these cells during this process has been reported to be a suitable marker for determining the degree of degranulation [3]. After an allergen triggers the allergic reactions, allergic mediators, including histamine, cytokines, and arachidonic acid derivatives, provoke acute and chronic allergic inflammation responses [4,5]. Various cells involved in the allergic reaction infiltrate the lesion. Among these, T helper 2 (Th2) cells are the most important cell type involved in atopic dermatitis development. Th2 cells release cytokines, such as IL-4, IL-5, and IL-13, in allergic inflammation and atopic dermatitis. The cytokines released by Th2 cells lead to the proliferation and activation of both mast cells and eosinophils in atopic and allergic skin inflammation, consequently leading to pruritus and impaired skin barrier function [6]. In particular, IL-4 contributes to the expansion of the Th2 cell subset from naïve T cells and the isotype switching of B cells to produce IgE against specific environmental allergens [7]. Cytokines, such as IL-4 and IL-5, are representative markers of the allergic reaction, based on their roles against allergens.

*Arctium lappa L*. is a popular edible vegetable cultivated in many countries. The roots are widely used in food, whereas the seeds are used in traditional medicine as diuretic, antipyretic, or detoxifying agents [8]. There are reports that *A. lappa *has anti-inflammatory [9], free radical scavenging [10], and antioxidant [11] activities, and that components [12] also have desmutagenic [13] and hepatoprotective [14] effects. Although *A. lappa *and its components have these biological activities, no reported study has evaluated the anti-allergic or anti-inflammatory effects of *A. lappa *root in atopic dermatitis or the molecular mechanisms involved. We examined the butanol extract of *A. lappa *(ALBE) roots because it significantly inhibited antigen-induced β-hexosaminidase release. Atopic dermatitis is a chronic, allergic inflammatory skin disorder, and we investigated both the anti-allergic and anti-inflammatory effects of ALBE. We examined the anti-allergic effects by checking the release of β-hexosaminidase induced by dinitrophenyl (DNP)-BSA in RBL-2H3 mast cells and expression levels of IL-4 and IL-5 in primary splenocytes after treatment with concanavalin A (ConA), which generates Th2 cytokines as in an allergic environment. We also examined the translocation of NF-κB and the phosphorylation of MAPKs, which are activated during inflammation, in ConA-treated primary murine splenocytes to validate the anti-inflammatory effects of ALBE.

## Methods

### Preparation of extract

Roots of *Arctium lappa L*. (1 kg) were extracted with 30% ethanol under reflux (10 L, 24 h, twice). The extract solutions were filtered and then evaporated at 40°C under reduced pressure, yielding 88.8 g of dry powder. Approximately 50 g of the ethanol extract were resuspended in 1 L of water and then partitioned with equal volumes of n-hexane, AcOEt, and n-BuOH to give n-hexane, AcOEt, n-BuOH, and H_2_O fractions. The butanolic fraction weighed 22.0 g and the sample was named *A. lappa *butanolic extract (ALBE).

### Cell culture and experimental animals

The RBL-2H3 rat mast cell line was obtained from the American Type Culture Collection (Rockville, MD, USA) and grown in minimum essential medium (MEM) with 15% fetal bovine serum (FBS), 2 mM L-glutamine, 100 U/mL penicillin, and 100 μg/mL streptomycin at 37°C in a humidified incubator with a 5% CO_2 _/95% air atmosphere. Specific-pathogen-free 8-10-week-old male C57BL/6 mice were purchased from Orient Bio (Gyeonggi-do, Korea) and housed in an animal room at a temperature of 23 ± 1°C and a humidity of 55 ± 5%, with a 12/12-h light/dark cycle. The mice were fed a standard laboratory diet with tap water *ad libitum*.

Animal care and all experimental protocols were performed following the Institute for Laboratory Animal Research (ILAR) guidelines.

### Materials

The anti-dinitrophenyl (DNP)-IgE and 4-nitrophenyl N-acetyl-β-D-glucosaminide were from Sigma-Aldrich, DNP-bovine serum albumin (BSA) was from Biosearch Technologies, minimum essential medium was from Invitrogen, fetal bovine serum (FBS) was from WelGENE, enzyme immunoassay reagents for cytokine assays, such as IL-4 and IL-5, were from BD Biosciences, the protein assay kit was from Bio-Rad Laboratories, anti-pERK, anti-ERK, anti-pJNK, anti-JNK, and anti-p-p38 were from Cell Signaling Technology, anti-p65 and anti-p38 were from Santa Cruz Biotechnology, anti-β-actin was from Sigma-Aldrich, anti-α-tubulin was from Abfrontier, the ECL chemiluminescence system was from GE Healthcare, and the polyvinylidene difluoride (PVDF) membrane was from Millipore. The polymerase chain reaction (PCR) oligonucleotide primers were custom synthesized by Bionics (Korea).

### XTT assay for cell cytotoxicity and proliferation

Splenocyte cytotoxicity and proliferation were examined using the XTT assay kit, according to the manufacturer's instructions. The spleen was removed aseptically and dissociated into a single cell suspension in culture medium. Cells (5 × 10^5 ^cells/well) were incubated with various ALBE concentrations (1, 10, 100 μg/mL) in the presence or absence of ConA at 3 μg/mL for T cell activation. After incubating the cells for 72 h, a mixture of 25 μL of phenazine methosulfate (PMS; electron-coupling reagent) and 25 μL of XTT [2,3-bis(2-methoxy-4-nitro-5-sulfophenyl)-2H-tetrazolium-5-carboxanilide] was added to each well. The cells were further incubated for 4 h to allow XTT formazan production. The absorbance was determined with a microplate reader at a test wavelength of 450 nm and a reference wavelength of 690 nm.

### β-Hexosaminidase release assay

Degranulation of RBL-2H3 cells was evaluated by measuring the activity of the granule-stored enzyme-β-hexosaminidase secreted in the extracellular medium. Cells were cultured in 24-well plates (2 × 10^5 ^cells/well) overnight. The cells were sensitized with anti-DNP-IgE (100 ng/mL) for 16 h at 37°C. After washing the cells with TGCM buffer (136 mM NaCl, 2.68 mM KCl, 0.36 mM NaH_2_PO_2_H_2_O, 1 mM CaCl_2_, 0.5 mM MgCl_2_, 11.9 mM NaHCO_3_, 5 mM dextrose, 1 g/L gelatin, pH 7.4), they were pretreated with ALBE (1, 10, 100 μg/mL) for 30 min and then treated with DNP-BSA (1 μg/mL) for 30 min at 37°C. Aliquots of the cellular supernatant (15 μL) were transferred to 96-well plates and incubated with 60 μL of substrate (1 mM *p*-nitrophenyl-N-acetyl-β-D-glucosaminide in citrate 0.05 M, pH 4.5) for 60 min at 37°C. The cells were lysed with 0.1% Triton X-100 before removing the supernatant to measure the total β-hexosaminidase activity. The reaction was stopped by adding 150 μL of Na_2_CO_3_-NaHCO_3 _buffer 0.1 M, pH 10. The absorbance at 405 nm was measured with a microplate reader (Themo Labsystems). The results were presented as the percentage of total β-hexosaminidase content of the cells determined by cell lysis with 0.1% Triton X-100.

$$\%\text{Degranulation}={\text{OD}}_{\text{supernatant}}/\left({\text{OD}}_{\text{supernatant}}+{\text{OD}}_{\text{triton x}-\text{1}00}\right)\times \text{1}00$$

### NA preparation and mRNA analysis by RT-PCR

Total splenocytes were plated at 3 × 10^7 ^cells/mL and treated with ALBE (100 μg/mL) and ConA (3 μg/mL) for 16 h. Total RNA from the treated cells was prepared with the TRIzol Reagent (Invitrogen), according to the manufacturer's protocol, and stored at -70°C until use. For detecting cytokines, including IL-4 and IL-5, total RNA was extracted after stimulation and treatment. The sequences of the primers used in this study were: IL-4 forward, 5'-ATG GGT CTC AAC CCC CAG CTA GT-3'; IL-4 reverse, 5'-GCT CTT TAG GCT TTC CAG GAA GTC-3'; IL-5 forward, 5'-AGC ACA GTG GTG AAA GAG ACC TT-3'; IL-5 reverse, 5'-TCC AAT GCA TAG CTG GTG ATT T-3'; GAPDH forward, 5'-GTG GCA AAG TGG AGA TTG TTG CC -3', and GAPDH reverse, 5'-GAT GAT GAC CCG TTT GGC TCC-3'. Each transcript was quantified as described in the instrument manual and normalized to the amount of GAPDH, a housekeeping gene.

### Measurement of cytokine production (IL-4 and IL-5 secretion)

For cytokine immunoassays, total splenocytes were plated at 3 × 10^7 ^cells/mL and treated with ALBE (100 μg/mL) and ConA (3 μg/mL) for 16 h. Culture supernatants were collected and the amount of secreted IL-4 and IL-5 was measured using an enzyme-linked immunosorbent assay (ELISA) using the protocol supplied by BD Biosciences.

### Subcellular fractionation

Cytosolic and nuclear extracts were prepared. In brief, splenocytes (5 × 10^7 ^cells/mL) were plated into 100-mm dishes and treated with ALBE (100 μg/mL) and ConA (3 μg/mL) for 4 h. The harvested cells were resuspended in 0.2 ml of buffer A (10 mM HEPES at pH 7.5, 1.5 mM MgCl_2_, 10 mM KCl, 1 mM DDT, 0.1% NP-40, 0.2 mM PMSF). The cells were lysed on ice for 15 min, and centrifuged (5,000*g*, 5 min, 4°C). The supernatant was collected as cytosolic extracts. The nucleic pellet was washed with buffer A lacking NP-40, and resuspended in 0.025 ml of buffer C (20 mM HEPES, pH 7.5, 25% glycerol, 0.42 M NaCl, 0.2 mM EDTA, 1.5 mM MgCl_2_, 1 mM DDT, 0.2 mM PMSF). After incubation on ice for 30 min, nuclear debris was spun down (13,000*g*, 10 min, 4°C). The supernatant was collected as nuclear extracts. The protein concentration was measured using a protein assay kit (Bio-Rad).

### Western blotting

Total splenocytes were plated at 3 × 10^7 ^cells/mL and treated with ALBE (100 μg/mL) and ConA (3 μg/mL) for 15 min and then harvested and lysed in a lysis buffer containing 20 mM Tris, pH 7.6, 150 mM NaCl, and 1% Triton X-100 with a protease inhibitor cocktail. Protein contents were measured using a protein assay kit (Bio-Rad). Samples were diluted with 1 × lysis buffer containing 1% β-mercaptoethanol. Equal amounts of cellular protein (50 μg) were resolved by 10% SDS-PAGE and transferred onto nitrocellulose membranes. After blocking, membranes were incubated with the target antibody and then with horseradish peroxidase-conjugated secondary antibody to IgG. Immunoreactive proteins were visualized using the ECL Western blot detection system. The protein level was compared to a loading control, such as β-actin or non-phosphorylated protein.

### Statistical analyses

Each experiment was repeated three or four times, and the results of a representative experiment are shown. The results are expressed as the means ± SEM and were compared using Student's *t*-test. A statistical probability of *p *< 0.05 was considered significant (# *p *< 0.05, ## *p *< 0.01, * *p *< 0.05, and ** *p *< 0.01).

## Results

### ALBE inhibits antigen-induced β-hexosaminidase release in IgE-sensitized mast cells

**Figure 1:**
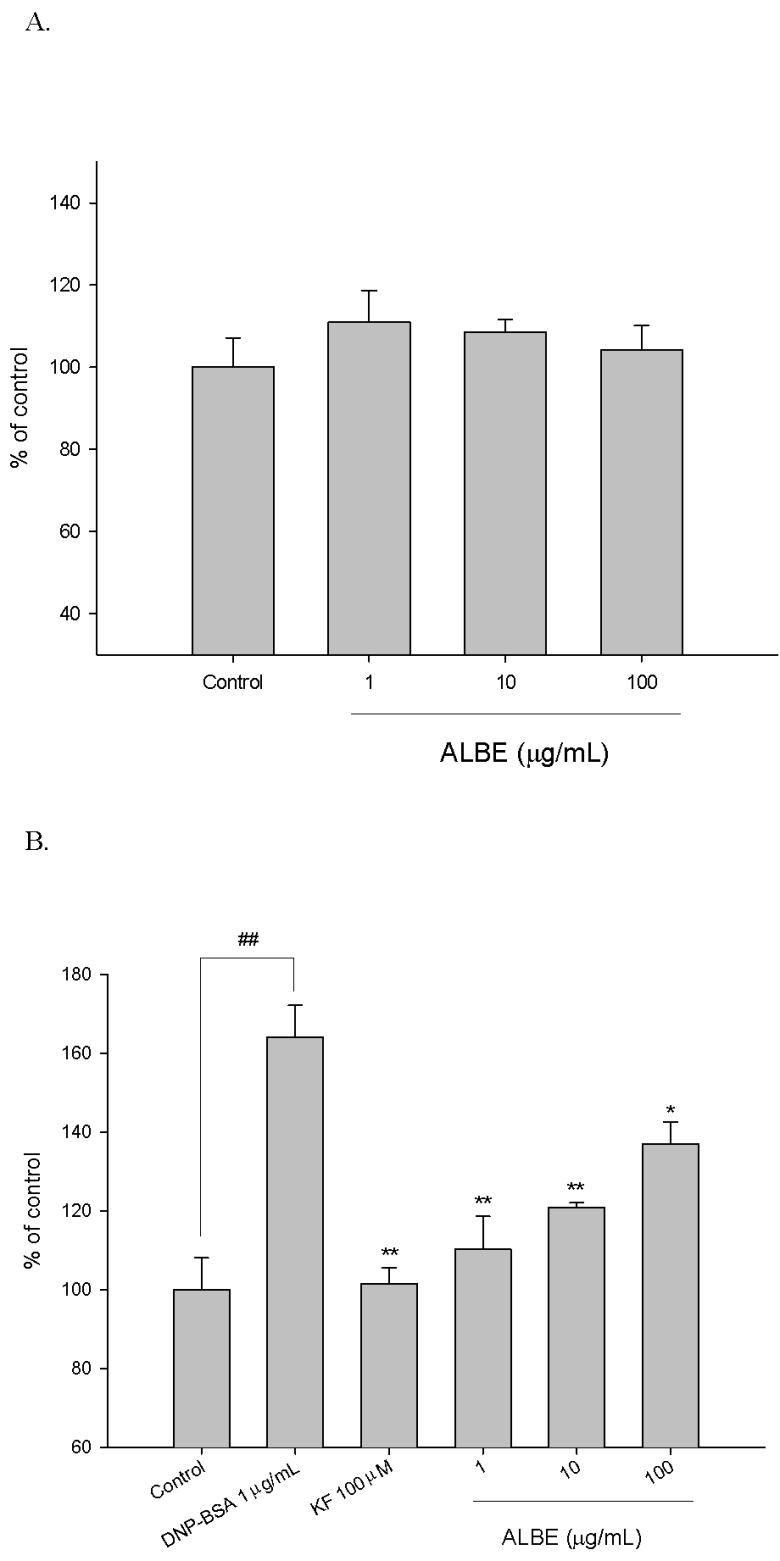
**Effects of ALBE on cell viability and antigen-induced β-hexosaminidase in RBL-2H3 cells**. (A) The cells were treated with various concentration of ALBE for 24 h. Cell viability was assessed using XTT assay. Absorbance was measure data at 450 nm and 650 nm. (B) The cells were sensitized by overnight incubation with 1 μg/ml of DNP-specific IgE in medium. This DNP-IgE-sensitized RBL-2H3 cells were pre-incubated with various concentration of ALBE for 30 min and then incubated with antigen (DNP-BSA) for 15 min in order to measure the release of β-hexosaminidase. Each bar shows the means ± SEM of four independent experiments. ^##^P < 0.01: significantly different from control group **P < 0.01, *P < 0.05: significantly different from DNP-BSA alone. KF; ketotifen fumarate.

Rat mast cell line RBL-2H3 cells were used to determine the effect of ALBE on the secretion of β-hexosaminidase. Initially, we measured the cytotoxicity of ALBE on RBL-2H3 cells using the XTT assay. ALBE at concentrations ranging from 1-100 μg/mL did not significantly affect the cytotoxicity in 24 h (Figure 1A). Thus, we treated DNP-IgE-sensitized RBL-2H3 cells with ALBE ranging from 1-100 μg/mL in subsequent experiments. ALBE significantly suppressed the DNP-BSA induced β-hexosaminidase secretion in IgE-sensitized RBL-2H3 cells at 1, 10, and 100 μg/mL and the effects are dose-dependent (Figure 1B). Ketotifen fumarate, an anti-allergic drug, also decreased the β-hexosaminidase secretion. The results showed that ALBE significantly inhibited antigen-induced mast cell degranulation.

### Effects of ALBE on cell proliferation and cytokine (IL-4, IL-5) secretion in ConA-induced primary murine splenocytes

**Figure 2:**
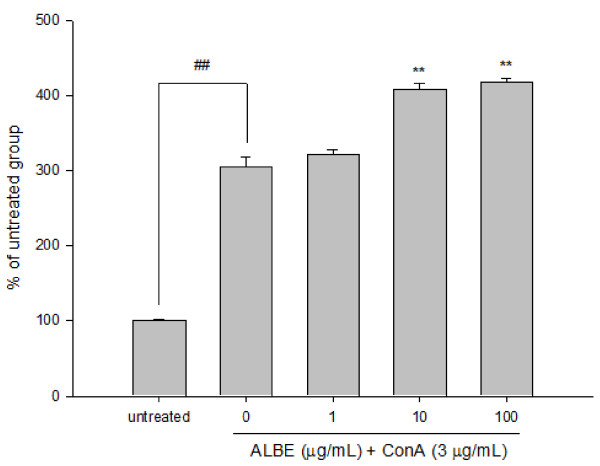
**Effects of ALBE on the proliferation of ConA-induced primary murine splenocytes**. Splenocytes were treated with various concentrations of ALBE and ConA (3 μg/ml) for 72 h. Cell proliferation was assessed using XTT assays. Absorbance was measure data at 450 nm and 650 nm. Each bar shows the means ± SEM of four independent experiments. ^##^P < 0.01: significantly different from the untreated group. **P < 0.01: significantly different from the ConA alone group.

**Figure 3:**
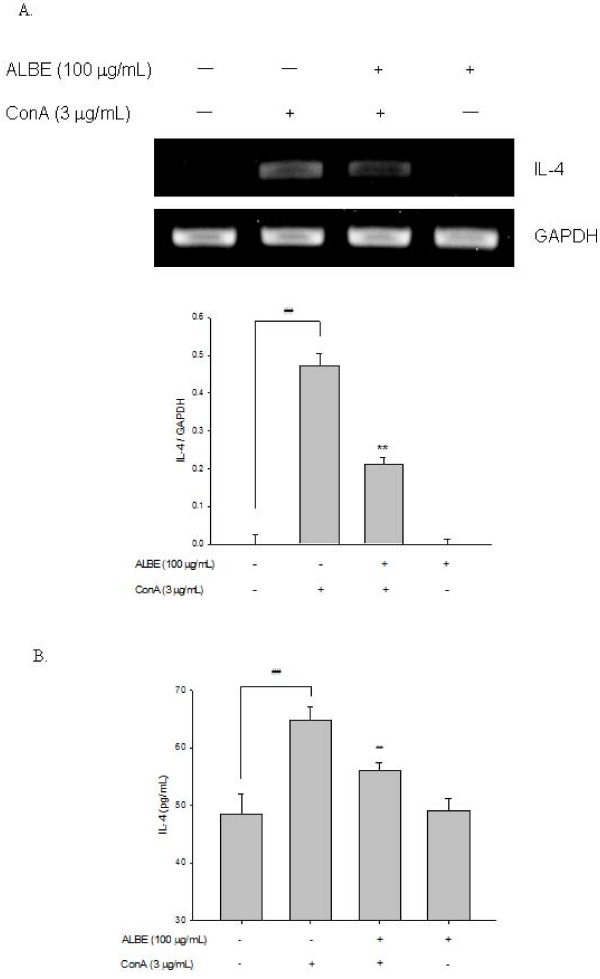
**The mRNA expressions and protein secretions of IL-4 by ALBE in primary murine splenocytes**. (A) The effects of ALBE on the mRNA expression of IL-4. ALBE (100 μg/ml) were treated to splenocytes with or without ConA (3 μg/ml) for 16 h. The mRNA expression of IL-4 was assessed by RT-PCR described in method. Each bar shows the means ± SEM of three independent experiments. (B) The effects of ALBE on the protein secretion of IL-4. ALBE (100 μg/ml) were treated to splenocytes with or without ConA (3 μg/ml) for 16 h. The protein secretion of IL-4 was assessed by ELISA described in methods. Each bar shows the means ± SEM of four independent experiments. ^##^P < 0.01: significantly different from the untreated group. **P < 0.01: significantly different from the ConA alone group.

**Figure 4:**
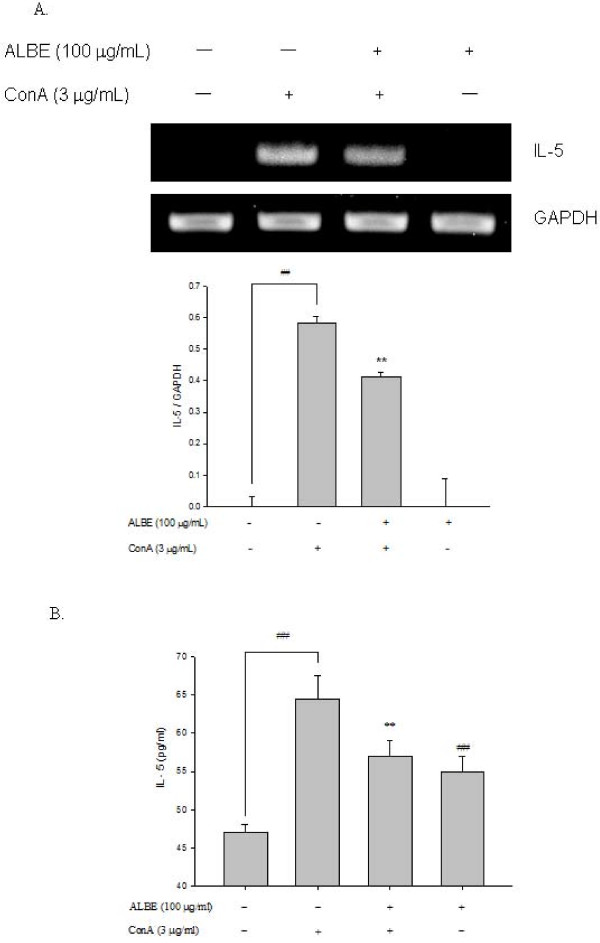
**The mRNA expressions and protein secretion of IL-5 by ALBE in primary murine splenocytes**. (A) The effects of ALBE on the mRNA expression of IL-5. ALBE (100 μg/ml) were treated to splenocytes with or without ConA (3 μg/ml) for 16 h. The mRNA expression of IL-5 was assessed by RT-PCR described in method. Each bar shows the means ± SEM of three independent experiments. (B) The effects of ALBE on the protein secretion of IL-5. ALBE (100 μg/ml) were treated to splenocytes with or without ConA (3 μg/ml) for 16 h. The protein secretion of IL-5 was assessed by ELISA described in methods. Each bar shows the means ± SEM of four independent experiments. ^##^P < 0.01: significantly different from the untreated group. **P < 0.01: significantly different from the ConA alone group.

We examined the effects of ALBE on ConA-induced T cell proliferation in primary murine splenocytes for 72 h to examine the immunomodulatory effect of ALBE. The concentration and duration of ALBE treatment without ConA had no effect on splenocyte viability (data not shown). As shown in Figure 2, ALBE significantly increased splenocyte proliferation in ConA-treated cells at 10 and 100 μg/mL (*p *< 0.05). Additionally, we examined the effects of ALBE on the expression and secretion of Th2 cytokines, such as IL-4 and IL-5, in primary murine splenocytes using RT-PCR and ELISA assays to investigate the further involvement of ALBE in Th2 functions in the atopic dermatitis-like skin lesions. ConA-induced IL-4 and IL-5 secretion was suppressed by ALBE treatment in splenocytes (Figure 3, Figure 4). ALBE treatment without ConA had no effect on IL-4 or IL-5 mRNA expression (data not shown), whereas ALBE with ConA significantly decreased the mRNA expression of IL-4 (to 55.3%) and IL-5 (to 29.0%) at 100 μg/mL, compared with ConA-stimulated splenocytes (Figure 3A, Figure 4A). In agreement with the RT-PCR results, ALBE inhibited the protein secretion of IL-4 (to 13.6%) and IL-5 (to 10.8%) under the same conditions (Figure 3B, Figure 4B). These results suggest that ALBE had immunostimulatory effects on T cells and meaningfully inhibited the antigen-induced mRNA expression and production of cytokines related to allergic and atopic reactions.

### Effects of ALBE on NF-κB activation and phosphorylation of MAPKs in ConA-induced primary murine splenocytes

**Figure 5:**
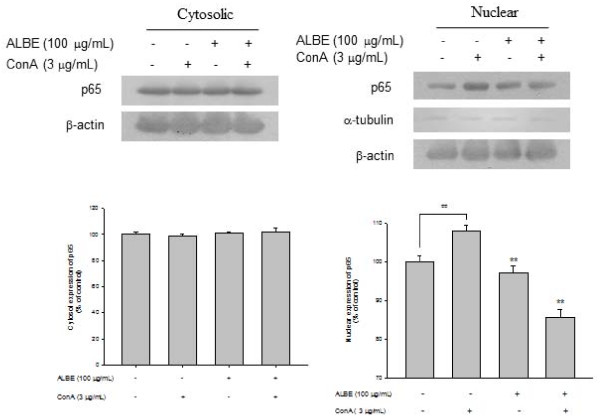
**Effects of ALBE on NF-κB activation in ConA-induced primary murine splenocytes**. ALBE (100 μg/ml) were treated to splenocytes with or without ConA (3 μg/ml) for 15 min. After isolation of cytosolic and nuclear fraction, the translocation of NF-κB (p65) was assessed by Western blotting described in methods respectively. ^##^P < 0.01: significantly different from the untreated group. **P < 0.01: significantly different from the ConA alone group.

**Figure 6:**
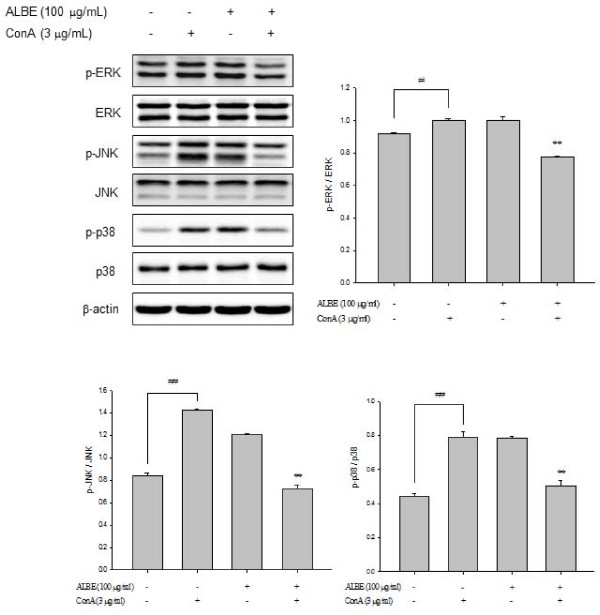
**Effects of ALBE on phosphorylations of p38 MAPK in ConA-induced primary murine splenocytes**. ALBE (100 μg/ml) were treated to splenocytes with or without ConA (3 μg/ml) for 15 min. The phosphorylations of p38 MAP kinase such as p38, JNK and ERK were assessed by Western blotting described in methods. ^#^P < 0.05, ^##^P < 0.01: significantly different from the untreated group. **P < 0.01: significantly different from the ConA alone group.

Increased expression of NF-κB (p65) was observed in the nucleus after treatment with ALBE plus ConA for 4 h (Figure 5). The relative intensity of NF-κB (p65) translocation in the nucleus was increased to 6.3% in the presence of ConA compared with the absence of ConA in the control. In contrast, the relative intensity of NF-κB (p65) translocation in the nucleus was decreased considerably, to 8.7%, after the addition of 100 μg/mL ALBE in the presence of ConA compared with ConA treatment alone. These data demonstrate that ALBE attenuated NF-κB activation and might affect downstream IL-4 and IL-5 production. ALBE inhibits ConA-induced phosphorylation of MAP kinases such as p38, JNK, and ERK (Figure 6). We found that ALBE attenuated not only the ConA-induced increase in the activity of NF-κB, but also the phosphorylation of MAPKs and these results suggest that ALBE may prevent allergic and atopic inflammation via NF-κB and the MAPKs signaling pathway.

## Discussion

Traditional medicines isolated from natural products often have positive effects in the prevention and healing of various immune disorders, such as allergy and atopic inflammation. In this study, the butanol fraction of *Arctium lappa L*. showed potential anti-allergic and anti-inflammatory effects by decreasing β-hexosaminidase release in mast cells and the secretion of IL-4 and IL-5 in ConA-induced T cells. Mast cells are primary effector cells involved in the allergic or immediate hypersensitivity responses [15]. The antigen crosslinking of the IgE-FcεRI complexes through the aggregation of IgE and FcεRI on mast cells results in the release of β-hexosaminidase, which is a marker of mast cell degranulation. The release of β-hexosaminidase and histamines also causes the production of proinflammatory cytokines, such as IL-4, IL-6, and TNF-α, which can potentiate inflammatory immune responses through the subsequent induction of other atopic inflammatory mediators. Thus, the modulation of cytokines in this process is considered a rational approach for regulating the early phase of allergic responses [5,15].

Atopic dermatitis is characterized by allergic skin inflammation. Pathological changes in atopic skin are observed as epidermal thickening and marked infiltration of inflammatory cells [16]. Atopic dermatitis has been associated with the Th2 phenotype and dominance of IL-4, IL-5, and IL-13 secretion [17,18]. We examined the inhibitory effects of ALBE on ConA-induced proliferation and cytokine (IL-4 and IL-5) secretion of splenocytes, which were used as a marker of Th2 lymphocyte function, to characterize the T cell immunomodulatory profile of ALBE. ALBE increased the ConA-induced proliferation and inhibitory effects on cytokine (IL-4 and IL-5) secretion in primary murine splenocytes. ALBE suppressed allergic-related Th2 function by decreasing the release of IL-4 and IL-5. However, it increased the total number of T cell subsets (Th1/Th2), indicating that it might decrease allergic-related Th2 cell function in some way without suppressing the immune system because it can augment all T cell subsets.

IL-4 acts as an eosinophil chemoattractant, which makes endothelial cells produce eosinophil chemotactic factor and eotaxin [19]. IL-4 is also essential in IgE production [20] and the switch from naïve T cells to allergic Th2 cells [21]. An immunohistochemical examination of the skin lesions in NC/Nga atopic model mice revealed the typical features of affected skin observed in patients with atopic dermatitis, such as increased infiltration of T cells, mast cells, and substantial expression of IL-4 and IL-5 [22,23]. That ALBE can decrease the secretion of IL-4 and IL-5 released by ConA-induced Th2 cells indicates that it might have a useful effect in allergic and atopic inflammation. We subsequently evaluated the related mechanisms of ALBE on cytokine secretion, including NF-κB activation and the phosphorylation of MAPKs. NF-κB is a key transcription factor that regulates the expression of genes involved in immune and inflammatory responses that require inflammatory cytokine production. NF-κB translocation and the MAPKs pathway are regarded as important processes in the regulation of the innate and acquired immune responses and chronic inflammation [24,25]. NF-κB is also a critical transcription factor that regulates Th2 cell differentiation and Th2-dependent airway inflammation [26].

We detected the inhibitory effects of ALBE on ConA-induced nuclear translocation of NF-κB (p65). Increased NF-κB activity has been reported in asthma, an allergic disease, and the inhibition of NF-κB activity decreased asthma [25]. Thus, we suggest that ALBE could have an anti-allergic effect based on the decrease in activated NF-κB it causes. Conventional MAP kinases are classified into three families: the c-Jun N-terminal kinases (JNKs), the p38 MAP kinases, and the extracellular signal-regulated kinases (ERKs). Intracellular signal transduction, including the phosphorylation of p38 MAPK, is subsequently followed by NF-κB translocation, leading to the production of cytokines and chemokines. We also showed that ALBE significantly suppressed the ConA-activated phosphorylation of p38 MAPK in primary murine splenocytes. It has been reported that p38 MAPK activation can activate transcription factors that result in the expression of IL-4, IL-5, and IL-13 in human T cells in response to antigen exposure in allergic disease [25]. The fact that ALBE decreased ConA-activated MAPKs and mRNA expression of IL-4 and IL-5 supports the possibility that ALBE may have anti-allergic and anti-inflammatory effects.

## Conclusions

ALBE may exert anti-allergic and anti-inflammatory activities by suppressing the transcription of NF-κB and the activated MAPKs signal pathway in splenocytes. Additionally, ALBE inhibited the antigen-induced degranulation of mast cells, as determined by the decreased release of β-hexosaminidase. From these results, we suggest that ALBE might be useful as a therapeutic agent for treating various forms of allergic inflammation, including atopic dermatitis.

## Competing interests

The authors declare that they have no competing interests.

## Authors' contributions

EHS carried out the molecular genetic studies, and drafted the manuscript. SAJ, SP, carried out the immunoassays and western blotting. HJ carried out the RT-PCR and XTT assay. SCK participated in the design of the study and performed the statistical analysis. CHL and SYK conceived of the study, and participated in its design and coordination and helped to draft the manuscript. All authors read and approved the final manuscript.
